# Ozempification of Aesthetics: Why Plastic Surgeons Must Lead the GLP-1 Era

**DOI:** 10.1093/asj/sjag060

**Published:** 2026-03-24

**Authors:** Johnny Franco

I applaud the authors for taking on this difficult and timely topic. GLP-1 medications have become one of the hottest topics of conversation not only in aesthetics, but throughout medicine and popular culture. More than 70% of Americans are currently overweight or obese—this affects a wide range of individuals either personally or through someone close to us.^1^ We have arrived at a pivotal moment in plastic surgery: we now have the ability to change our patients’ lives in a way that was never previously possible.

Plastic surgery has always been about form and function. Now we have the opportunity, and I would argue, the obligation, to help guide our patients to both look and live better. Few medications in modern medicine have created a ripple effect across health, wellness, and aesthetics the way GLP-1 and dual GIP/GLP-1 therapies have over the past several years. What we are experiencing is more than a trend; it is a profound shift at the intersection of aesthetics, wellness, longevity, and traditional medicine—with the single goal of enhancing our patients’ lives.

This junction has created polarizing views with respect to where the world of aesthetics should be involved in GLP-1 therapy, which sits at the crossroads of health, wellness, aesthetics, and beauty. Those categories are no longer separate in the minds of our patients, and they should not be separate in the minds of plastic surgeons either. What used to be fragmented is now a continuum. This expanding spectrum of treatment options is exciting and challenging. It is unquestionable that the use of GLP-1 medications comes with responsibility. We have an ethical obligation to our patients and to our specialty to educate our patients and ourselves. Plastic surgeons have always led the way in innovation and the evolution of aesthetic interventions, and the future of aesthetics will be intimately intertwined with wellness and longevity. “Look good, feel good” will no longer be a catchy statement; it will be something we can provide, guide, and build with our patients over time.

Throughout the history of our specialty, we have been trained to optimize patients medically and surgically. From the burn unit to critical care in the ICU to complex reconstruction, plastic surgeons are not strangers to maximizing a patient's health prior to intervention. The shift now is that optimizing health is becoming part of the aesthetic intervention itself. Our specialty is evolving, and in the not-too-distant future we will either lead the movement of building beauty from the inside out or we will be left behind as this will continue to be a larger part of the aesthetic journey in the future.

The concept of the “Ozempification of aesthetics” developed as I watched how GLP-1 medications such as Ozempic, Mounjaro, and others changed our specialty so rapidly. Currently 1 in 8 Americans are on a GLP-1 medication.^2^ There is no question this has created opportunity, with many patients now entering the aesthetic arena for the first time. It has created a category of aesthetic-naïve patients who will be introduced to the aesthetic world due to the secondary effects of these GLP-1 medications.^3^ However, this incredible influx of new patients into the aesthetic arena comes with an obligation to help guide them on their journey. There will be an entirely new host of challenges as we see young patients with mature issues presenting to our offices in unprecedented numbers. The weight-loss journey, particularly rapid medically driven weight loss, is complex. Waiting on the periphery while patients undergo their weight-loss journey and subsequent changes will, in my opinion, lead to the increased need for intervention later. We don’t have to just accept these changes. We can participate in the journey from day one to minimize unintended aesthetic effects from these medications.

**Figure 1:**
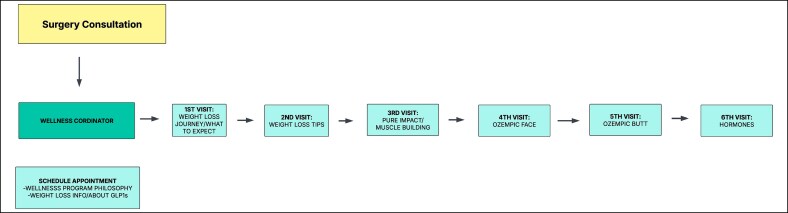
Weight Loss Journey - Educational Drip Campaign

This is why our practice has evolved to incorporate a more active role in the journey, and it is important to understand that this can be done whether or not you are prescribing the medication. Prevention of “Ozempic face” begins the moment a patient starts their weight-loss journey. Patients deserve to understand risks and benefits just as they would with any medical or aesthetic treatment—but we do not have to stand idle on the sidelines and accept volume loss, skin changes, and muscle changes as inevitable consequences. We are in a unique position to minimize, and in some cases prevent, these changes. From the outset, patients should have a comprehensive plan, curated by us, for their life-changing journey. In our practice we have a specific journey that our patients are guided through over the first year. (Figure 1). That does not mean we should not incorporate other specialists when appropriate; it means we remain intimately involved from start to finish.

The current GLP-1 medications are just the beginning of the weight-loss medications. Evolving peptides and therapies are entering the wellness and longevity world that we will need to lead. We are a specialty built on evolution and innovation—we have always guided patients to be the best version of themselves. We should embrace the wellness wave, educate ourselves, and become a resource and leader in this next era of aesthetics. Multidisciplinary care is valuable and absolutely has a place in complex situations, and that is no different than what we do today.^4^ If a patient is already on diabetic medication, they should be referred back to their endocrinologist to evaluate whether a GLP medication is appropriate. I am not suggesting plastic surgeons treat diabetes or become their primary care physician, however—the majority of patients that present to our office can be safely treated by well-trained plastic surgeons and their aesthetic team.

The day is coming, and in many practices is already here, when discussing modern body sculpting will require discussing GLP medications as part of the treatment spectrum along with liposuction, tummy tuck, and fat transfer. Visceral fat is something we have never been able to meaningfully impact as plastic surgeons. That is no longer true in the modern era of GLP-1 medications. GLP-1 treatments will increasingly become part of the overall aesthetic plan—not as a replacement for surgery, but as a tool that helps patients reach aesthetic and wellness goals that are becoming inseparable.

There are currently over 50 GLP-1 type medications in clinical trials and over 300 GLP-1 type medications in some stage of discovery.^5^ This pipeline of medications reinforces that this is not a passing trend, but will be an integral part of our specialty for years to come. The future will hold a spectrum of therapies tailored to maximize patient health, wellness, and aesthetics by addressing muscle preservation, visceral fat, obesity, inflammation, and beyond. AbbVie, the parent company of Allergan, for example, has signed a licensing agreement to develop an amylin-based obesity drug.

The old model of “lose weight and come back when you’re ready” failed patients and typically was not productive for the providers. Historically, many patients were told to lose weight before surgery and return when they reached a certain BMI or weight. Too often, they were left discouraged and overwhelmed, and the majority never returned. Many never lost weight and many never improved their overall health. GLP-1 medications have changed that dynamic—we no longer have to send patients away. The paradigm has shifted from triage to guiding.

I understand the hesitation in aesthetics around prescribing and managing GLP-1 medications, especially as we all believe in the ethical principle of “do no harm.” That principle is ingrained in all of us, and I agree with it completely. It means using therapies responsibly, understanding risks, selecting patients appropriately, and monitoring outcomes. Multiple studies have supported the safety of GLP-1 medications and that is one reason they have become widely used and accepted. Specifically, in patients without diabetes the risk of severe adverse outcomes is extremely rare.^6^ The risk of hypoglycemia in this group has been reported as less than 1%.^7^ Beyond safety, evidence continues to grow on meaningful health benefits, including reduction in major adverse cardiovascular events in the SELECT trial.^8^ It is unquestionable that every medication should be used with caution. We must understand side effects and risk. But we also must understand what the data say about safety and benefits of these treatments. We must be involved in the conversation.

What I have seen in our practice is that most patients are not pursuing GLP-1 therapy solely for cosmetic surgery. They are trying to reclaim energy, confidence, and longevity. And when they feel better, they start showing up differently in life. That is why I believe plastic surgeons should be involved in this journey not to replace primary care or endocrinology, but to complement them and bridge the gap between our various specialties as many patients have been lost in the nebulous gap over the past few years. When done responsibly, that involvement includes appropriate patient selection, clear referral pathways when complexity is high, longitudinal planning with a 12- to 24-month roadmap that includes weight targets, protein and strength guidance, skin health, and timing for procedures, perioperative coordination including anesthesia planning, symptom screening, and medication timing, and ethical boundaries built on informed consent, realistic expectations, and avoiding “quick-fix” marketing.

To be transparent, I am personally invested in this conversation. I have been on my own weight-loss journey and have experienced first-hand how these medications can change not just appearance, but quality of life. That perspective is part of why I integrated this into our practice. It has been the single best thing I have done for myself and arguably one of the most impactful things we have implemented for our patients. The weight-loss revolution and GLP-1 medications are not a trend. This is a shifting paradigm. It has changed how we view weight in America, changed the conversation, and expanded treatment options for patients who are overweight or obese.

We are entering a wellness era in aesthetics. Plastic surgery has become more than removing fat, tightening skin, and excising tissue. It has become about a patient journey that revolves around an entire transformation from the inside out. For many patients, GLP-1 therapy is the start of that journey, often a journey they did not believe was possible. I agree with the authors that if plastic surgeons are going to be part of the GLP-1 journey, we must be educated, responsible, and continually up to date. But I would go one step further: given how deeply this impacts surgical planning, outcomes, and long-term outcomes, and given what it means for the future of our specialty, we should not be peripheral voices in this discussion. We should be leading it, alongside our patients.
